# Cytisus scoparius link - A natural antioxidant

**DOI:** 10.1186/1472-6882-6-8

**Published:** 2006-03-16

**Authors:** Raja Sundararajan, Nazeer Ahamed Haja, Kumar Venkatesan, Kakali Mukherjee, Bishnu Pada Saha, Arun Bandyopadhyay, Pulok Kumar Mukherjee

**Affiliations:** 1 School of Natural Product Studies, Department of Pharmaceutical Technology, Jadavpur University, Kolkata 700 032, India; 2 Molecular Endocrinology Laboratory, Indian Institute of Chemical Biology, Kolkata 700 032, India

## Abstract

**Background:**

Recent investigations have shown that the antioxidant properties of plants could be correlated with oxidative stress defense and different human diseases. In this respect flavonoids and other polyphenolic compounds have gained the greatest attention. The plant *Cytisus scoparius *contains the main constituent of flavone and flavonals. The present study was undertaken to evaluate the *in vitro *antioxidant activities of extract of aerial part of *Cytisus scoparius*.

**Methods:**

The plant extract was tested for DPPH (1, 1-diphenyl, 2-picryl hydrazyl) radical scavenging, nitric oxide radical scavenging, superoxide anion radical scavenging, hydroxyl radical scavenging, antilipid peroxidation assay, reducing power and total phenol content.

**Results:**

The extract exhibited scavenging potential with IC_50 _value of 1.5 μg/ml, 116.0 μg/ml and 4.7 μg/ml for DPPH, nitric oxide and superoxide anion radicals. The values were found to lesser than those of vitamin C, rutin, and curcumin, as standards. The extract showed 50% protection at the dose of 104.0 μg/ml in lipid peroxidation induced by Fe^2+^/ ascorbate system in rat liver microsomal preparation. There is decrease in hydroxyl radical generation with IC_50 _value of 27.0 μg/ml when compared with standard vitamin E. The reducing power of the extract depends on the amount of extract. A significant amount of polyphenols could be detected by the equivalent to 0.0589 μg of pyrocatechol from 1 mg of extract.

**Conclusion:**

The results obtained in the present study indicate that hydro alcoholic extract of aerial part of *Cytisus scoparius *is a potential source of natural antioxidants.

## Background

Oxidative stress plays an important role in the pathogenesis of various diseases such as atherosclerosis, alcoholic liver cirrhosis and cancer etc [1,2]. Oxidative stress is initiated by reactive oxygen species (ROS), such as superoxide anion (O ^- ^_2_), perhydroxy radical (HOO^-^) and hydroxyl radical (HO^·^). These radicals are formed by a one electron reduction process of molecular oxygen (O_2_). ROS can easily initiate the lipid peroxidation of the membrane lipids, causing damage of the cell membrane of phospholipids, lipoprotein by propagating a chain reaction cycle [3]. Thus, antioxidants defense systems have coevolved with aerobic metabolism to counteract oxidative damage from ROS. Most living species have efficient defense systems to prevent themselves against oxidative stress induced by ROS [4]. Recent investigations have shown that the antioxidant properties of plants could be correlated with oxidative stress defense and different human diseases like aging process etc [5-8]. In this respect flavonoids and other polyphenolic compounds have received the greatest attention [9].

The genus *cytisus *consists of about 70 species confined to the mild climate regions of south and central Europe, North Africa and West Asia. About four species have seen introduced into India and one of them *Cytisus scoparius *Link (Family – Leguminosae) is now fairly common in the Nilgiri and Simla hills. In ethnomedical information, this plant used for diuretics, hypnotic & sedative [10], diabetes [11] and liver disease [12]. Pharmacological studies have confirmed its uterine stimulant effect [13] and anti spasmodic activity [14]. It has also been found to have diuretic activity, hypotensive activity [14] and estrogenic effect. The plant *Cytisus scoparius *contains the flavone such as 6'' O acetyl scoparin [15], flavonals namely rutin, quercetin, quercitrin, isorhamnetin and kaempferol [16] and some isoflavones like genistein and sarothamnoside [17]. Quinolizidine alkaloids namely spartein, sarothamine and lupanine [18] and Benzenoid compounds like tyramine, hydroxyl tyramine [19], phenyl ethanol and cresol [20] have also been reported to be present in the plant. Most of the reported biological activities and active constituents of this plant may be related to its antioxidant nature. Based on this idea the *in vitro *antioxidant activity of the extracts of aerial part of *Cytisus scoparius *has been evaluated and reported hereunder.

## Methods

### Chemicals

Rutin was obtained from Acros organics, New Jersy, USA. DPPH (1, 1-diphenyl, 2-picryl hydrazyl), NBT (Nitro blue tetrazolium), NADH (Nicotinamide adenine dinucleotide phosphate reduced), PMS (Phenazine methosulphate), TCA (Trichloro acetic acid), Ferric chloride and BHT (Butylated hydroxy toluene) were obtained from Sigma chemical co USA. Ascorbic acid, curcumin and Vitamin E were obtained from SD Fine chem. Ltd, Biosar, India. TBA (Thiobarbituric acid) and pyridine were obtained from Loba chemie, Mumbai, India. EDTA (Ethylene diamine tetra acetic acid) and Hydrogen peroxide (H_2_O_2_) were obtained from Qualigens Fine chemicals, Mumbai, India. Naphthyl ethylene diamine dihydrochloride was obtained from Roch-light ltd, Suffolk, England. Sodium nitro prusside was obtained from Ranbaxy lab, Mohali, India. Pottassium ferric cyanide was obtained from May and Backer, Dagenham, UK. 2-deoxy-2- ribose was obtained from Fluka (Buchs, Switzerland).

### Plant material

Aerial parts of *Cytisus scoparius *was collected in Nilgiri hills, Tamilnadu region and authenticated through Government Arts College, Ooty. Voucher specimen (SNPS-011/ 2003–2004) of this plant material has been retained in the School of Natural Product Studies, Jadavpur University, India.

### Extraction

The aerial part of *Cytisus scoparius *plant was dried at room temperature and reduced to coarse powder. This powder was extracted with mixture of ethanol: water (7:3 ratio) for 48 hours. The solvent was completely removed by rotary evaporator and further removal of water was carried out by freeze drying. The dried extract was stored in vacuum desiccator for further use.

### DPPH radical scavenging activity

The free radical scavenging capacity of the extracts was determined using DPPH. A methanol DPPH solution (0.15%) was mixed with serial dilutions (0.5 μg to 8 μg) of *Cytisus scoparius *extracts and after 10 min, the absorbance was read at 515 nm using a spectrophotometer (Perkin – Elmer Lambda 20 UV – visible spectrophotometer). Vitamin C used as a standard. The inhibition curve was plotted and IC_50 _values obtained [21].

### Nitric oxide radical inhibition assay

Nitric oxide radical inhibition can be estimated by the use of Griess Illosvoy reaction [22]. In this investigation, Griess Illosvoy reagent was modified by using naphthyl ethylene diamine dihydrochloride (0.1% w/v) instead of 1-napthylamine (5%). The reaction mixture (3 ml) containing sodium nitroprusside (10 mM, 2 ml), phosphate buffer saline (0.5 ml) and *Cytisus scoparius *extract (10 μg to 160 μg) or standard solution (rutin, 0.5 ml) was incubated at 25°C for 150 min. After incubation, 0.5 ml of the reaction mixture mixed with 1 ml of sulfanilic acid reagent (0.33% in 20% glacial acetic acid) and allowed to stand for 5 min for completing diazotization. Then, 1 ml of naphthyl ethylene diamine dihydrochloride was added, mixed and allowed to stand for 30 min at 25°C. A pink coloured chromophore is formed in diffused light. The absorbance of these solutions was measured at 540 nm against the corresponding blank solutions. Rutin used as a standard.

### Superoxide anion scavenging activity

Measurement of superoxide anion scavenging activity of *Cytisus scoparius *was done based on the Nishimiki method [23]. About 1 ml of nitroblue tetrazolium (NBT) solution (156 μM NBT in 100 mM phosphate buffer, pH 7.4) 1 ml NADH solution (468 μM in 100 mM phosphate buffer, pH 7.4) and 0.1 ml of sample solution of *Cytisus scoparius *(1.25 μg to 10 μg) in water were mixed. The reaction started by adding 100 μl of phenazine methosulphate (PMS) solution (60 μM PMS in 100 mM phosphate buffer, pH 7.4) to the mixture. The reaction mixture was incubated at 25°C for 5 min, and the absorbance at 560 nm was measured against blank samples. Decreased absorbance of the reaction mixture indicated increased superoxide anion scavenging activity. Curcumin was used as a positive control.

### Lipid peroxidation assay

The rat liver microsomal fraction was prepared by the method of Bouchet et al [24]. The reaction mixture contained in a final volume of 1.0 ml, 500 μl of liver microsomal fraction, 300 μl buffer containing the plant extract (50–150 μg), 100 μl of FeCl_3 _(1 mM) and 100 μl ascorbic acid (1 mM) to start peroxidation. Samples were incubated at 37°C for 1 hour, after that lipid peroxidation was measured using the reaction with thiobarbituric acid (TBA). Thiobarbituric acid reactive substances were determined by the methods of Houghton and Aruoma [25,26]. The absorbance of the organic layer was measured at 532 nm. All reactions were carried out in triplicate. Vitamin E used as a standard.

### Hydroxyl radical scavenging assay

The assay was performed as described by Halliwell method [27] with minor changes. All solutions were prepared freshly. 1.0 ml of the reaction mixture contained 100 μl of 28 mM 2-deoxy-2-ribose (dissolved in phosphate buffer, pH 7.4), 500 μl solution of various concentrations of the *Cytisus scoparius *(10 to 80 μg), 200 μl of 200 μM FeCl_3 _and 1.04 mM EDTA (1:1 v/v), 100 μl H_2_O_2 _(1.0 mM) and 100 μl ascorbic acid (1.0 mM). After an incubation period of 1 hour at 37°C the extent of deoxyribose degradation was measured by the TBA reaction [23,24]. Measure the absorbance at about 532 nm against the blank solution. Vitamin E was used as a positive control.

### Reducing power

The reducing power of *Cytisus scoparius *was determined according to the Oyaizu method [28]. Different concentration of *Cytisus scoparius *extract (100 μg – 1000 μg) in 1 ml of distilled water was mixed with phosphate buffer (2.5 ml, 0.2 M, pH 6.6) and potassium ferricyanide [K_3_Fe(CN)_6_] (2.5 ml, 1%). The mixture was incubated at 50°C for 20 min. A portion (2.5 ml) of trichloroacetic acid (10%) was added to the mixture, which was then centrifuged at 3000 rpm for 10 min. The upper layer of the solution (2.5 ml) was mixed with distilled water (2.5 ml) and FeCl_3 _(0.5 ml. 0.1%) and the absorbance was measured at 700 nm. Increased absorbance of the reaction mixture indicated increased reducing power. Butylated hydroxy toluene used as a standard.

### Determination of total phenolic compounds

Total soluble phenolic in the aqueous extract of *Cytisus scoparius *were determined with Folin-Ciocalteu reagent according to the standard method [29] using pyrocatechol as a standard. Briefly, 0.1 ml of extract solution (contains 1000 μg extract) in a volumetric flask diluted distilled water (46 ml). About 1 ml of Folin – Ciocalteu reagent was added and the contents of the flask mixed thoroughly. After 3 min, 3 ml of Na_2_Co_3_(2%) was added, then the mixture was allowed to stand for 2 hour with intermittent shaking. The absorbance was measured at 760 nm. The concentration of total phenolic compounds in the *Cytisus scoparius *determined as microgram of pyrocatechol equivalent by using an equation that was obtained from Gulcin et al [30]. The equation is given below:

Absorbance = 0.001 x Pyrocatechol (μg) + 0.0033

### Statistical analysis

All the invitro experimental results were mean ± S.D of five parallel measurements.

## Results

### DPPH radical scavenging activity

**Figure 1:**
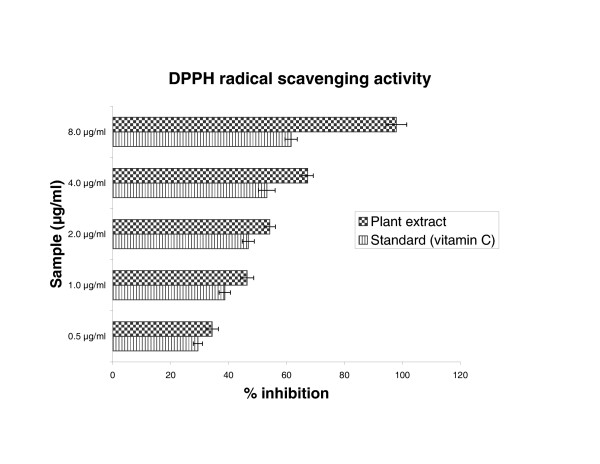
Scavenging effect of *Cytisus scoparius *extract and standard vitamin C on 1, 1'-Diphenyl-2-picryl hydrazyl (DPPH) radical. Results are mean ± S.D of five parallel measurements.

The hydro alcoholic extract of *Cytisus scoparius *exhibited a significant dose dependent inhibition of DPPH activity, with a 50% inhibition (IC_50_) at a concentration of 1.5 μg/ml. The result was mentioned in figure 1. The IC_50 _value of the extract was found to be lesser than the standard, vitamin C (IC_50 _3.0 μg/ml).

### Nitric oxide radical inhibition assay

**Figure 2:**
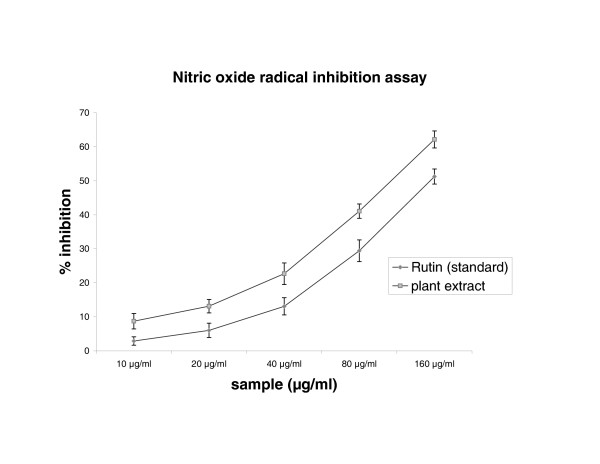
Scavenging effect of *Cytisus scoparius *extract and standard rutin on Nitric oxide radical. Results are mean ± S.D of five parallel measurements.

The scavenging of nitric oxide by plant extract was increased in a dose-dependent manner as illustrated in figure 2. At concentration of 116.0 μg/ml of extract 50% of nitric oxide generated by incubation was scavenged. This IC_50 _value of extract found to be lesser than the standard, rutin (IC_50 _160.0 μg/ml).

### Superoxide anion scavenging activity

**Figure 3:**
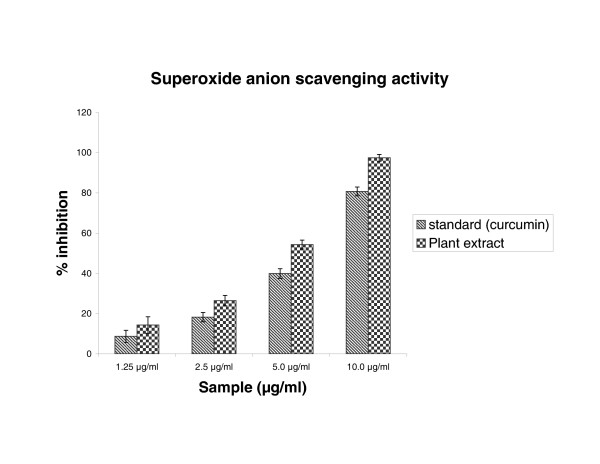
Effect of *Cytisus scoparius *extract and curcumin on scavenging of superoxide anion radical formation. Results are mean ± S.D of five parallel measurements.

The superoxide anion derived from dissolved oxygen by Phenazine methosulphate/NADH coupling reaction reduces nitro blue tetrazolium. The decrease the absorbance at 560 nm with the plant extract thus indicates the consumption of superoxide anion in the reaction mixture. As mentioned in figure 3, the plant extract as well as curcumin showed the scavenging activity; IC_50 _values, 4.7 μg/ml and 5.84 μg/ml, respectively.

### Lipid peroxidation assay

**Figure 4:**
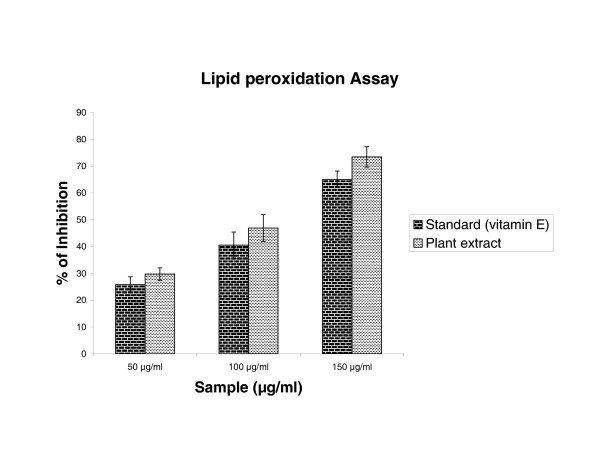
Effect of *Cytisus scoparius *extract and vitamin E on lipid peroxidation of liver microsome induced by Fe^2+^/ascorbate. Results are mean ± S.D of five parallel measurements.

Activity of plant extract against non-enzymatic lipid peroxidation in rat liver microsomes has been shown in figure 4. Addition of Fe ^2+^/ascorbate to the liver microsomes cause increase in lipid peroxidation. The extract showed inhibition of peroxidation effect in all concentrations, which showed 50% inhibition effect at 104.0 μg/ml. The extract inhibition value was found to be lesser than the standard, vitamin E (IC_50 _120.5 μg/ml)

### Hydroxyl radical scavenging assay

**Figure 5:**
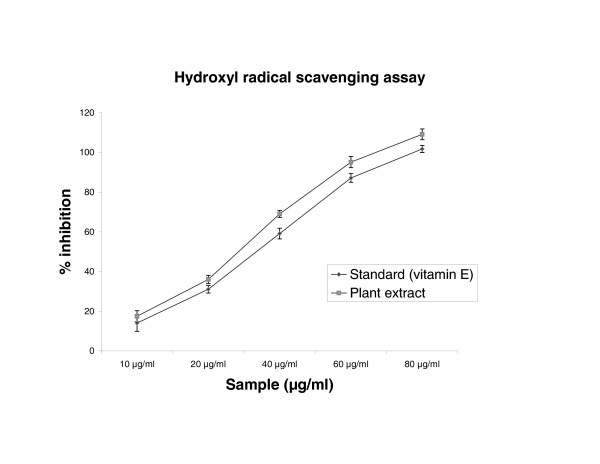
Effect of *Cytisus scoparius *extract and vitamin E on deoxyribose degradation assay. Results are mean ± S.D of five parallel measurements.

To attack the substrate deoxyribose hydroxyl radicals were generated by reaction of Ferric-EDTA together with H_2_O_2 _and ascorbic acid. When the plant extract were incubated with the above reaction mixture, it could prevent the damage against sugar. The results are shown in figure 5, the concentrations of 50% inhibition were found to be 27.0 μg/ml and 32.5 μg/ml for the extract and standard of vitamin E, respectively. The extract inhibition value was found to be lesser than the standard

### Reducing power

**Figure 6:**
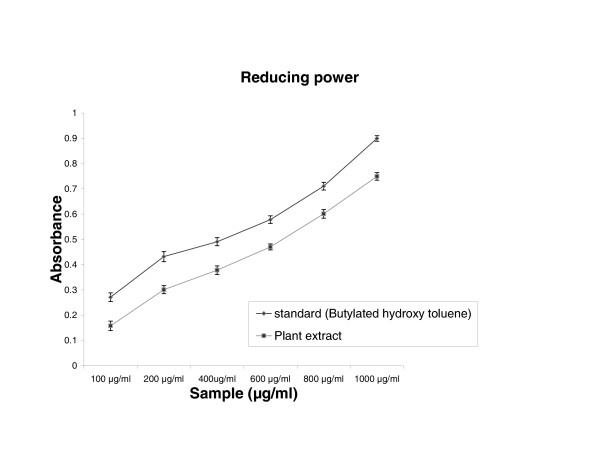
The reductive ability of *Cytisus scoparius* extract and butylated hydroxy toluene. Results are mean ± S.D of five parallel measurements.

Figure 6 shows the reductive capabilities of the plant extract compared to butylated hydroxy toluene. The reducing power of extract of *Cytisus scoparius *was very potent and the power of the extract was increased with quantity of sample. The plant extract could reduce the most Fe^3+ ^ions, which had a lesser reductive activity than the standard of butylated hydroxy toluene.

### Determination of total phenolic compounds

The total phenolic contents of hydro alcoholic extract of *Cytisus scoparius *was 0.0589 μg pyrocatechol equivalent /mg.

## Discussion

Free radicals have aroused significant interest among scientists in the past decade. Their broad range of effects in biological systems has drawn the attention of many experimental works. It has been proved that these mechanisms may be important in the pathogenesis of certain diseases and ageing. There are many reports that support the use of antioxidant supplementation in reducing the level of oxidative stress and in slowing or preventing the development of complications associated with diseases [31]. Many synthetic antioxidant components have shown toxic and/or mutagenic effects, which have shifted the attention towards the naturally occurring antioxidants. Numerous plant constituents have proven to show free radical scavenging or antioxidants activity [32]. Flavonoids and other phenolic compounds (hydroxyl cinnamic derivatives, catechines etc) of plant origin have been reported as scavengers and inhibitors of lipid peroxidation [33].

In our present study demonstrated that, DPPH is a free radical, stable at room temperature, which produces a purple colour solution in methanol. It is reduced in the presence of an antioxidant molecule, giving rise to uncoloured methanol solutions. Figure 1 illustrates the decrease in the concentration of DPPH radical due to scavenging ability of hydro alcoholic extract of plant and vitamin C, which is comparable to the reported value of Thabrew et al [34]. Nitric oxide radical inhibition study proved that aerial part of the extract is a potent scavenger of nitric oxide. This nitric oxide generated from sodium nitro prusside reacts with oxygen to form nitrite. The extract inhibits nitrite formation by competing with oxygen to react with nitric oxide directly and also to inhibit its synthesis. Scavengers of nitric oxide compete with oxygen leading to reduced production of nitric oxide [35]. From the nitric oxide test, rutin was used as a standard. The IC_50_ value of the rutin is comparable to the reported value of Badami et al [36].

In the PMS/NADH -NBT system, superoxide anion derived from dissolved oxygen by PMS/NADH coupling reaction reduces NBT. The decrease of absorbance at 560 nm with antioxidants thus indicates the consumption of superoxide anion in the reaction mixture. Addition of various concentrations of extract as well as curcumin (standard) in above coupling reaction showed decrease in absorbance. The antioxidant property of curcumin is generally attributed to its phenolic nature [37]. Sreejayan and Rao et al [38] have earlier observed that for superoxide and DPPH scavenging, the order of activity was: curcumin > demethoxycurcumin > bisdemethoxycurcumin > diacetylcurcumin (almost inactive). The liver microsomal fraction undergoes rapid non-enzymatic peroxidation when incubated with FeCl_3 _and ascorbic acid. The use of Fe (III) in the presence of a reducing agent such as ascorbate produces .OH [39] and they attack the biological material. This leads to the formation of MDA (malonodialdehyde) and other aldehydes, which form a pink chromogen with TBA, absorbing at 532 nm [40]. The extract and vitamin E exhibited strong scavenging effect of hydroxyl radical which could inhibit lipid damage at different concentration. The scavenging effect of vitamin E is in accordance with the report of Hemanth et al [41]. The extract was examined for its ability to act as .OH radical scavenging agent. Ferric EDTA was incubated with H_2_O_2 _and ascorbic acid at PH -7.4; hydroxyl radicals were formed in free solution and were detected by their ability to degrade 2-deoxy-2- ribose into fragments that on heating with TBA at low pH form a pink chromogen [26,27]. When *Cytisus scoparius *plant extract and vitamin E were added to the reaction mixture they removed hydroxyl radicals and prevented the degradation of 2-deoxy-2- ribose as mentioned above. The observed IC_50 _values of the extract and Vitamin E were analogous to the reported values of Sen et al [42]. Figure 6 shows the reductive capabilities of plant extract compared with butylated hydroxy toluene. For the measurements of the reductive ability, we investigated the Fe^3+ ^to Fe^2+ ^transformation in the presence of hydro alcoholic extract using the method of Oyaizu et al [28]. The reducing power increased with increasing the amount of extract. The reducing capacity of compound may serve as a significant indicator of its potential antioxidant activity [43]. The absorbance values of the extract at different concentrations were found to be less than that of the reference compound. The value of reference compound is in accordance with the report of Illhami et al [44]. The phenolic compounds may contribute directly to anti oxidative action [45]. This result indicates that polyphenol present in aerial part and its extract could be partly responsible for the beneficial effects. Compelling evidence indicates that increased consumption of dietary antioxidants or fruits and vegetables with antioxidant properties may contribute to the improvement in quality of life by delaying onset and reducing the risk of degenerative diseases associated with aging. Therapeutic potentials of various Indian medicinal plants of Simla hills are well documented from its traditional origin in different aspects [46,47]. These plant species require to be further explored for the possible molecular mechanisms which are underway at our laboratory.

## Conclusion

This study suggested that the *Cytisus scoparius*. L plant extract possess antioxidant activity, which might be helpful in preventing or slowing the progress of various oxidative stress- related diseases. Further investigation on the isolation and identification of antioxidant component(s) in the plant may lead to chemical entities with potential for clinical use.

## Competing interests

The author(s) declare that they have no competing interests.

## Authors' contributions

SR: Performed the study

KFHN: Performed the study

VK: Performed the study

KM: Design, analysis and acquisition of data

BPS: Helped to perform the study

AB: Helped to perform the study

PKM: Supervised the study design along with drafting the manuscript.

## Pre-publication history

The pre-publication history for this paper can be accessed here:


